# Metal-free Ternary BCN Nanosheets with Synergetic Effect of Band Gap Engineering and Magnetic Properties

**DOI:** 10.1038/s41598-017-07143-6

**Published:** 2017-07-26

**Authors:** Changlong Sun, Fukun Ma, Liang Cai, Aizhu Wang, Yongzhong Wu, Mingwen Zhao, Wensheng Yan, Xiaopeng Hao

**Affiliations:** 1 State Key Lab of Crystal Materials, Shandong University, 250100 Jinan, Shandong P.R. China; 2 National Synchrotron Radiation Laboratory, University of Science and Technology of China, 230029 Hefei, Anhui P.R. China; 3 Department of Physics, Shandong University, 250100 Jinan, Shandong P.R. China

## Abstract

Introducing the synergy effect of magnetic properties and band gap engineering is highly desired for two-dimensional (2D) nanosheets. Here, we prepare metal-free ternary 2D carbon (C) doped boron nitride (BN) nanosheets (BCN) with band gap engineering and magnetic properties by a synergetic way. The substitutional occupation of C atoms, as revealed by X-ray absorption spectrum, in BCN nanosheets induces tunable band gap reduction (5.5 eV to 2.6 eV) and intensive intrinsic ferromagnetism at room temperature. First-principle calculations also reveal that substituted C atoms in BCN nanosheets can broaden the light adsorption region and reduce the optical band gap, and ferromagnetic ordering is energetically more favorable than antiferromagnetic. This design opens up new possibility for synergetic manipulation of exchange interactions and band gap engineering in 2D nanostructures.

## Introduction

In recent years, metal-free two-dimensional (2D) nanosheets, such as graphene, have been widely studied due to their unique properties and wide range of applications. However, with the development of the nanotechnology^1, 2^, spontaneous magnetization has been confirmed by theoretically and experimentally in some 2D materials^3–11^. In contrast to the conventional magnetic materials that contain transition metal (*TM*) with 3*d* or 4 *f* electrons, the magnetism in these metal-free 2D systems originates from the light elements that involve only *s* and *p* electrons. Nevertheless, the intrinsic zero band gap and the diamagnetic nature of graphene limit their applications in spin-based multifunctional devices. As a graphene analogue, hexagonal boron nitride nanosheets (BNNSs) have received enormous attention due to their novel properties and possible applications in nanoelectronics^12^, but its large band gap (~6 eV) and diamagnetic nature also restricted its application^13^. Thus, band gap engineering and inducing stable ferromagnetism (*FM*) are equally important for BNNSs. Until now, *FM* has been achieved experimentally in BNNSs through defects^14^ or dopants^15^ control. However, to the best of our knowledge, there are no reports on the demonstration of controlling the band gap and optical properties of metal-free element doped BNNSs.

As primary doping elements for the origin of magnetism, the introduced *TM* atoms tend to cluster with each other because of the strong *d-d* interactions, which results in inhomogeneous distribution of the *TM* atoms^16, 17^. To date, research on *TM* elements doped BNNSs is limited to theoretical calculation^18, 19^. Hence, doping light or similar atomic radii elements which contain only *s* and *p* electrons are one of the most prospective methods to obtain magnetism. According to the theoretical calculation, the electron spin orientation can be changed and magnetic coupling can be easily introduced in carbon (C) doped BN^20^. In 2001, Okada *et al*. predicted that the ground state of B-N-C nanosheets with particular stoichiometry is ferromagnetic by the first-principles calculations in the density-functional theory^21^. Previous theoretical studies have also shown that C doped *h*-BN tubules facilitate magnetic ordering^22^. Choi *et al*. reported that periodic arrangements of heterojunctions in C doped *h*-BN systems can lead to the formation of a 1D itinerant ferromagnetic state^23^. Meanwhile, early theoretical studies have anticipated that the electronic structure and band gap of B-C-N system can be tuned depending on the composition and atomic arrangement of B, C and N^24^. Very recently, the weak *FM* has been observed in C-doped BNNSs which is ascribed to the C dopants^15^. However, the *FM* is very weak (~10^−3^ emu/g) and can be removed by oxidation treatment. Meanwhile, it is not very certain what the band gap change is when different C doped BNNSs (BCN). Hence, at present, realizing stable magnetic and tunable band gap in metal-free BCN nanosheets remains challenging in experiments, especially the synergetic effect between band gap engineering and *FM*.

In this contribution, we present a simple but efficient approach for achieving BCN nanosheets. We demonstrate for the first time that the incorporation of C atoms can not only induce remarkable band gap reduction but also intensive *FM* which is quite stable at high temperature and robust against oxidation. The saturation magnetization of the BCN nanosheets can reach 0.134 emu/g, which is comparable to defective or doped graphene^3, 4^. With the increase of C concentration, the band gap can be tuned from 5.5 eV to 2.6 eV accompanied by the great improvement of *FM*. The roles of C dopants in the interesting properties of BCN nanosheets that are distinct from both BNNSs and graphene are revealed using first-principles calculations. Our results not only open up new possibilities for manipulation of *FM* in metal-free nanomaterials but also provide a new candidate for optoelectronic devices.

## Results

**Figure 1:**
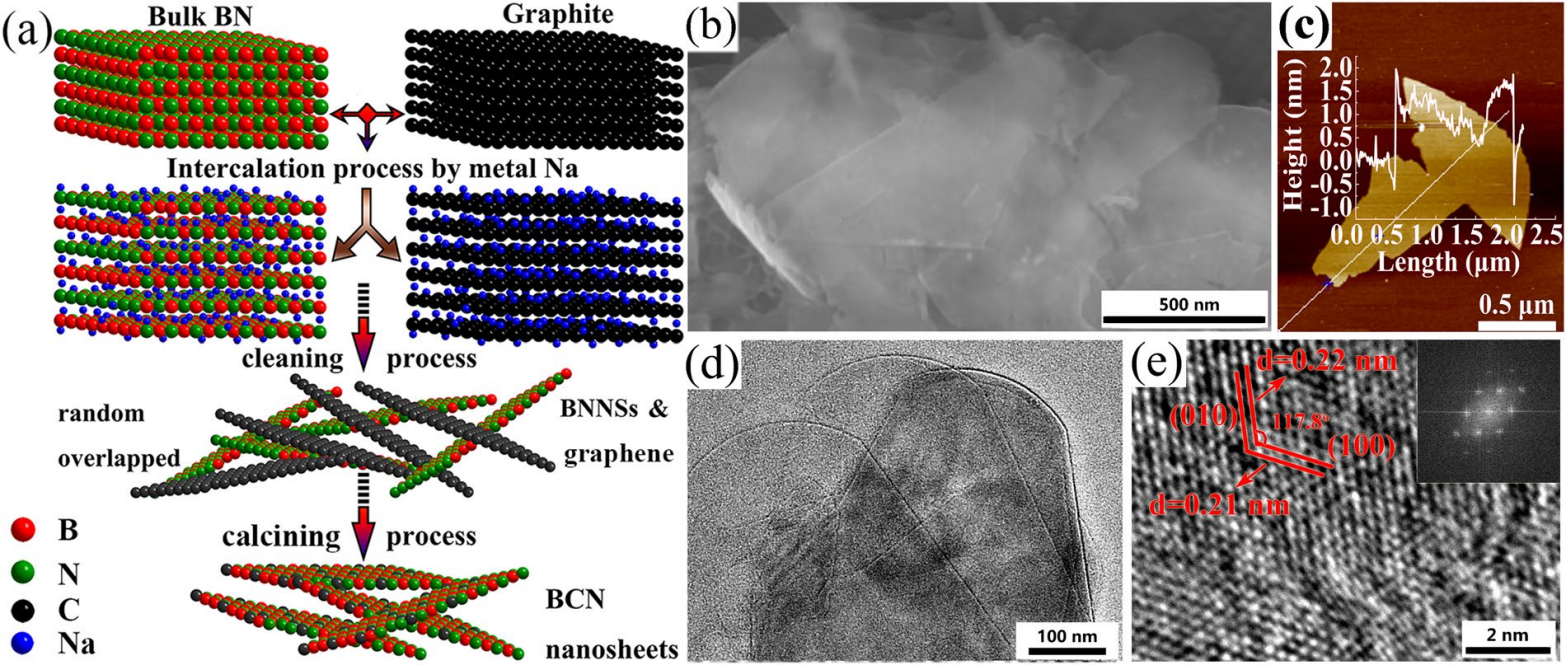
(**a**) Schematic of the processing steps involved in the synthesis of well-dispersed BCN nanosheets. (**b**) SEM image of the as-prepared BCN nanosheets. (**c**) AFM image of the as-prepared BCN nanosheets. (**d**) TEM image of a stack of thin flakes of as-prepared BCN nanosheets. (**e**) HRTEM image of the as-prepared BCN nanosheets; the inset shows the FFT image.

To successfully obtain the carbon doped BN, it is critical to control the thermal degradation behaviors. Slow increase of temperature guarantees that carbon species are chemically active enough to be doped into the lattice of host BN through a slow C/BN substitution reaction process^25^. The BCN nanosheets are synthesized by embedding C atoms into the lattice of BNNSs (Fig. 1a). A more detailed description of the experimental procedures is provided in the supporting information (S2). According to previous report, Na intercalation compounds react more violently with water than Li compounds, implying that nanosheets should be exfoliated more efficiently^26^. Additionally, the high chemical potentials of molten Na can insert into the interlayer space^27^, and the continuous hydration (ethanol) of the intercalated ions, leading to further lattice expansion^26^. The as-prepared BCN nanosheets are almost transparent even at low accelerating voltages, Fig. 1b, indicating the ultrathin nature of the sheets. As shown in Supplementary Fig. 1, BNNSs and graphite can be exfoliated well by metallic sodium and preserved for more than three months, respectively. BCN nanosheets were then calcined in open air to remove the surplus C species. The atomic force microscopy (AFM) image (Fig. 1c) shows that the BCN nanosheets are wrinkle-free and approximately 1.5 nm in thickness, ~1.5 μm in size. The high-resolution transmission electron microscopy (HRTEM) image (Fig. 1e) indicates that the basic structure of the BCN nanosheets remain intact. Corresponding interlayer spacing of the (010) and (100) planes is 0.22 nm and 0.21 nm, respectively. Notably, the angle between the (100) and (010) planes, 117.8°, is similar with the pristine BNNSs. A typical hexagonal symmetry can be observed in the fast Fourier transformation (FFT) images (insets of Fig. 1e) which show the single-crystalline nature of the BCN nanosheets.

**Figure 2:**
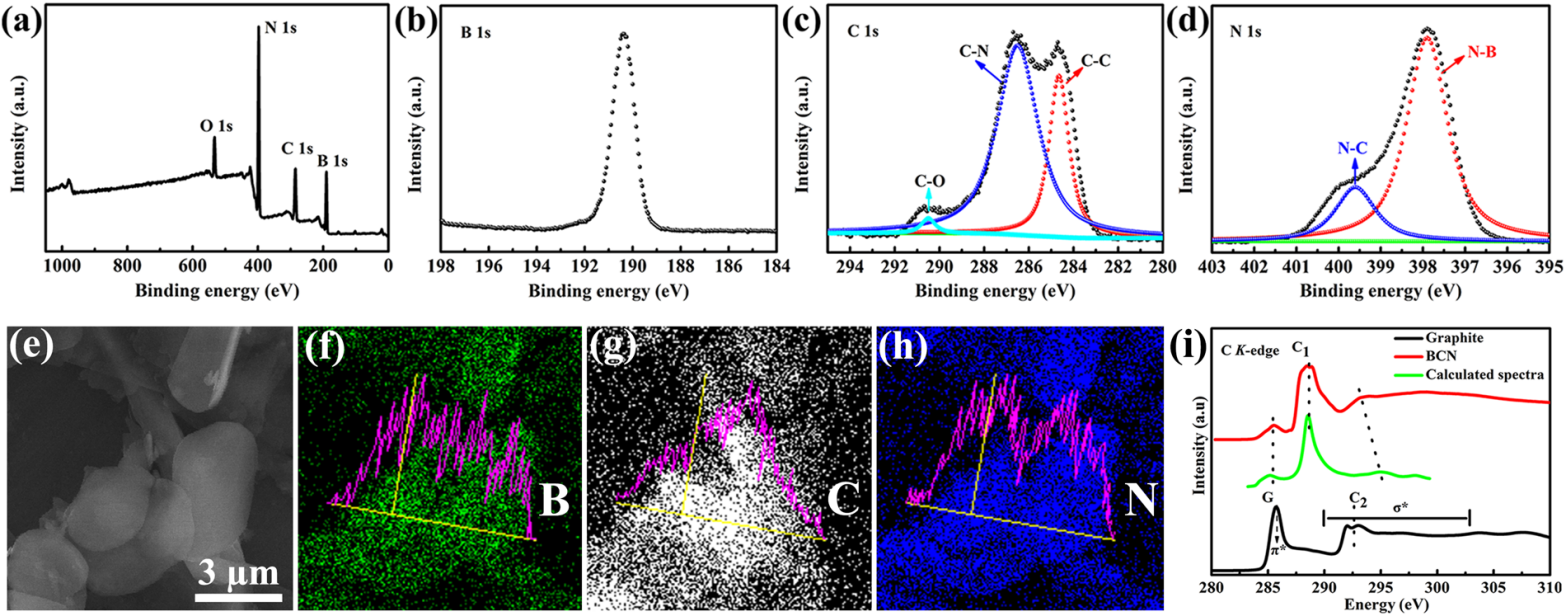
X-ray photoelectron spectroscopy (XPS) spectra and energy dispersive X-ray spectrum (EDS) element mapping of the as-prepared BCN nanosheets. (**a**) XPS survey spectrum of the as-prepared BCN nanosheets and high-resolution XPS spectra of (**b**) B1s, (**c**) C1s, and (**d**) N1s electrons. (**e**–**h**) SEM image and corresponding EDS element mapping of B, C, and N. (**i**) A comparison of C *K*-edge X-ray absorption near-edge structure (XANES) spectrum of BCN nanosheets and graphite.

X-ray photoelectron spectroscopy (XPS) was implemented to further study the composition and construction of the samples. Figure 2a shows the survey spectrum of the BCN nanosheets includes the peaks of B 1 *s*, C 1 *s*, N 1 *s*, and O 1 *s*, which is consistent with the EDS mapping results. The signal of C in BNNSs can be ascribed to the C contamination (Supplementary Fig. 2a). As shown in Fig. 2b, the typical high-resolution XPS spectrum of B 1 *s* at 190.3 eV is assigned to the B=N bonds in the BCN nanosheets, which is very close to that of B 1 *s* (190.1 eV) in BNNSs^28^. The C 1 *s* peak (Fig. 2c) can be fitted into three peaks: 284.6 eV, 286.5 eV, and 290.5 eV which correspond to the graphitic carbon (C=C)^29^, C=N^30^, and C−O bands, respectively. The peak at 286.5 eV can be attributed to the sp^2^ C atoms bonded to N in an aromatic ring. The subpeak at 290.5 eV (C−O bands) is due to the potential surface impurity^28^. The sp^2^ C atoms bonded to N in an aromatic ring (C=N, 286.5 eV) verify the successful C incorporation rather than surface-adsorbed C^31^. This scenario suggests the existence of C−N bonds, thus verifying successful C incorporation rather than surface-adsorbed C. In the high-resolution XPS spectrum of the N 1 *s* region (Fig. 2d), the peak at 397.9 eV is similar to the position of the N 1 *s* spectrum (398.1 eV) of BNNSs. However, because C atoms have a higher electronegativity than B^32^, a distinct shoulder at a higher energy (399.6 eV) implies a contribution from N atoms trigonally bonded with sp^2^ or sp^3^ C atoms^31^. The existence of bonding configurations of C and N from these XPS spectra suggests that the B, C, and N elements have a real ternary bonding nature in the hexagonal lattice. However, C−N bonds do not appear in the high-resolution XPS spectrum of the N 1 *s* region in the as-prepared BNNSs, as shown in Supplementary Fig. 2b. Furthermore, the SEM image and corresponding EDS mapping analyses reveal the homogeneous distribution of B, C, and N in all the ultrathin nanosheets. Comparing the X-Ray diffraction (XRD) results, the (002) peak of the as-obtained BCN nanosheets (Supplementary Fig. 3) was puny, indicating the ultrathin nature of these nanosheets. Meanwhile, compared with BNNS, the diffraction peaks of BCN nanosheets slightly shift to small angle. The chemical structure was further characterized with Fourier transform infrared (FT-IR, Supplementary Fig. 4). Two typical absorption peaks can be observed from both BCN nanosheets and BNNSs. Indeed, additional bands in our study due to carbon incorporation are assigned to C−N bonds (981.7 cm^−1^)^33^. However, due to the typically overlap with B–N bands, the C–N bonds located at 1200~1500 cm^−1^ cannot be detected, this is also consistent with the previous studies^29^.

In order to determine the local structure of the doped C atoms, the C *K*-edge X-ray absorption near-edge structure (XANES) spectrum of BCN nanosheets was compared with the graphite. As shown in Fig. 2i, the C *K*-edge XANES spectrum of graphite exhibits the peak (~285.5 eV, labeled as G) in the graphite π* region lines up with the π* feature, which indicates the presence of graphite-like *sp* ^2^-bonded carbon atoms. The feature at ~293 eV (C_2_) in the σ* region (290–303 eV) corresponds to transitions from the C1s to the σ* states^34^. However, the C *K*-edge XANES spectrum of BCN and graphite have quite different XANES features with a characteristic peaks (C_1_) at 288.5 eV that are primarily associated with the C-N bond. The calculated spectrum of the BCN with B atom replaced by C atom can reproduce the characteristic peaks (C_1_) of the experimental XANES spectrum of the BCN, and this also indicates the substitutional occupation of C dopants. The distinct spectral features could be reproduced by XANES calculations using FEFF8.2 code^35^ (see the details of structure model configurations and calculations in S1 in the Supporting Information).

Elemental analysis by XPS revealed that the C content is gradually increasing with increasing amounts of graphite. Thus, XPS analysis very clearly supports the incorporation of C in the BNNSs. Three different compositions of BCN (BCN-1, BCN-2, BCN-3, the C compositions determined from the XPS spectra are 5.8 (±0.1) %, 12.5 (±0.1) %, and 23.6 (±0.2) %, respectively) were prepared in the present study. No evidence of any other elements except B, C and N was found from the XPS measurements. In particular, both the XPS and ICP results (Table S1) exclude the existence of magnetic impurities.

**Figure 3:**
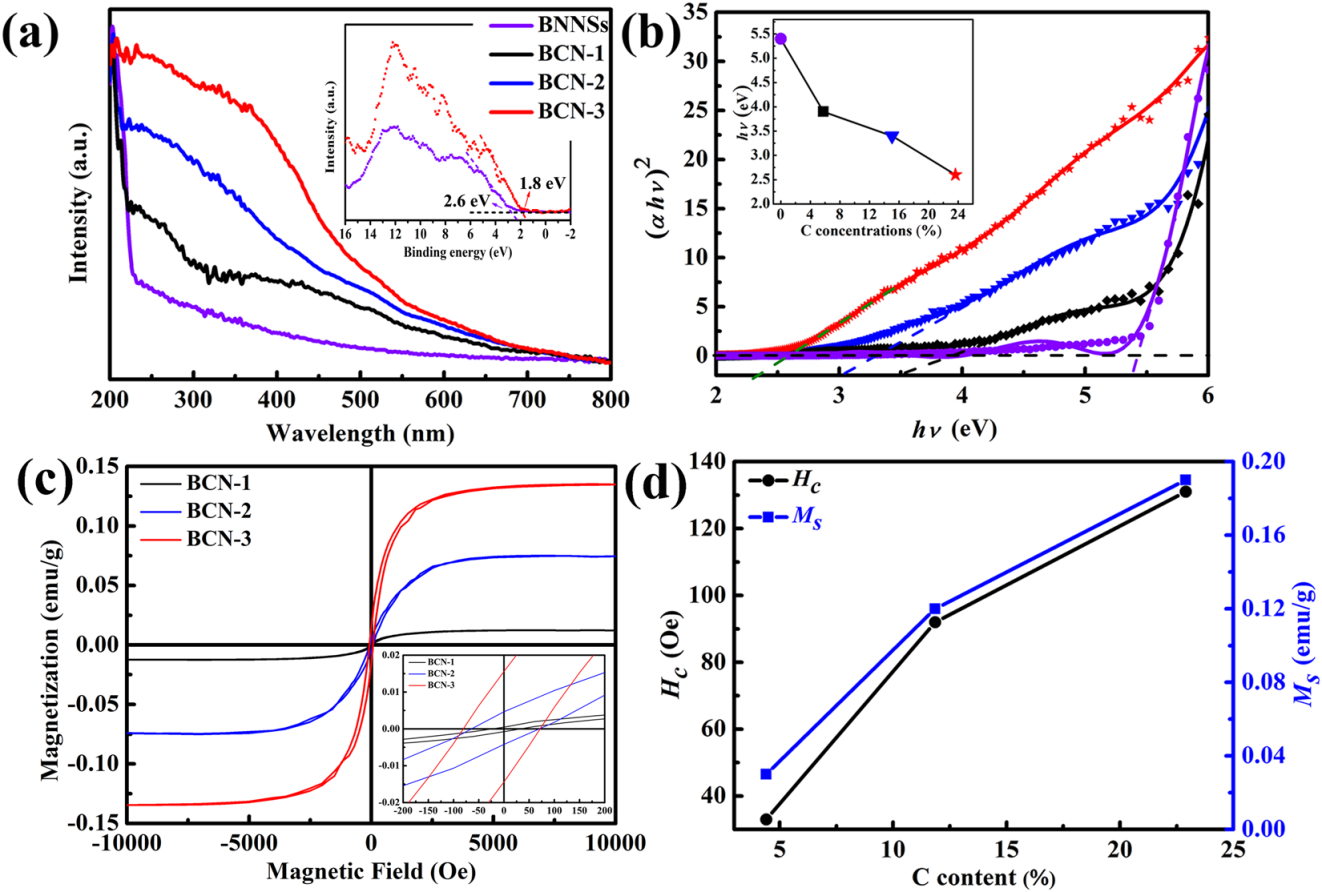
Absorption and magnetic properties of the as-prepared BCN nanosheets. (**a**) UV-vis diffuse reflectance spectra (DRS) results. Inset is the valence-band XPS spectra comparison of the BNNSs and BCN nanosheets. (**b**) Tauc plot of the as-prepared BCN nanosheets with different C concentrations. The inset shows the band gap variation of BNNSs and BCN nanosheets from the Tauc plot. (**c**) Magnetization vs. magnetic field (*M-H*) curves of different C concentration nanosheets measured at room temperature. The curves show nonzero magnetization at room temperature. (**d**) Relationship between saturation magnetization and coercive fields with C concentration.

The gray colour of the BCN nanosheets (Supplementary Fig. 5) which differ significantly from the raw materials strongly suggests the enhanced light absorption. The light adsorption and the optical energy gap properties of these materials are investigated using the UV-vis diffuse reflectance spectra (DRS). As shown in Fig. 3a, the as-prepared BCN nanosheets exhibit completely different light absorption from BNNSs and exfoliated graphite (more details in Supplementary Fig. 6a and b). Compared with BNNSs, the absorption in the visible light region is greatly enhanced. The absorption edge of the BCN nanosheets was red shifted and enhanced with the increase of C concentration. The long absorption tails in the visible indicates the presence of intraband impurity transitions. A similar phenomenon has occurred in the h-BCN prepared by pyrolysis method^29^. The optical band gap of BCN nanosheets can be estimated from the Tauc plot formulation^36^. For a direct band gap material, the curve of converted (*αhλ*)^2^ versus *hλ* from the UV-vis spectrum was utilized to determine the optical band gap of the BCN nanosheets^29^. Thus, the remarkably enhanced visible light absorption of the BCN nanosheets are resulted from the narrowed band gap^37^. With the increase of C concentration (Fig. 3b), the band gap of the BCN nanosheets can be tuned from 5.5 eV to 2.6 eV, which is comparable to the graphene-like 2-D layered transition metal dichalcogenides, such as MoS_2_ (~1.8 eV)^38^ and WS_2_ (~2.1 eV)^39^. From the valence band XPS (VBXPS) spectra, inset of Fig. 3a, the substantially blue-shifted VBM of BCN nanosheets further confirms the narrowed band gap of the BCN nanosheets^37^, which should be related to the substituted C atoms. However, unlike the h-BNC film prepared by chemical vapour deposition (CVD), the as-prepared BCN nanosheets is the substituted C doped rather than hybrid atomic layers of BNNSs and graphene^40^. As revealed by first-principles calculations discussed in the following sections, the enhanced light adsorption in the visible region and the optical band gap reduction can be ascribed to the change of the electronic states of the BCN nanosheets.

Figure 3c shows the isothermal magnetization *vs*. magnetic field (*M-H*) measured at room temperature (300 K) in the range of −10k Oe < *H* < 10k Oe. The diamagnetic contribution from the capsule and sample holder was mathematically subtracted to obtain a single contribution from BCN. All the BCN samples with different C contents exhibit ferromagnetic hysteresis loop (Fig. 3c). The well-defined hysteresis loops strongly suggest that the BCN nanosheets are ferromagnetic at least up to 300 K, which indicates that the ferromagnetic order dominates the entire BCN nanosheets with different stoichiometry. For comparison, the *M−H* curve of the pristine BNNSs and exfoliated graphite at 300 K is also shown (Supplementary Fig. 6c and d). The weak hysteresis loops indicate weak *FM* behavior of both samples, which is close to the previous reports^3, 14^. Figure 3d shows that saturation magnetization (*M*_*s*_) of BCN nanosheets are 0.004, 0.068, and 0.134 emu/g (0.12 *μ*_B_/C); the corresponding coercive fields (*H*_*C*_) are 25, 61, and 81 Oe, respectively. *M*_*s*_ and the corresponding *H*_*C*_ show an upward trend with the increase in C concentration.

**Figure 4:**
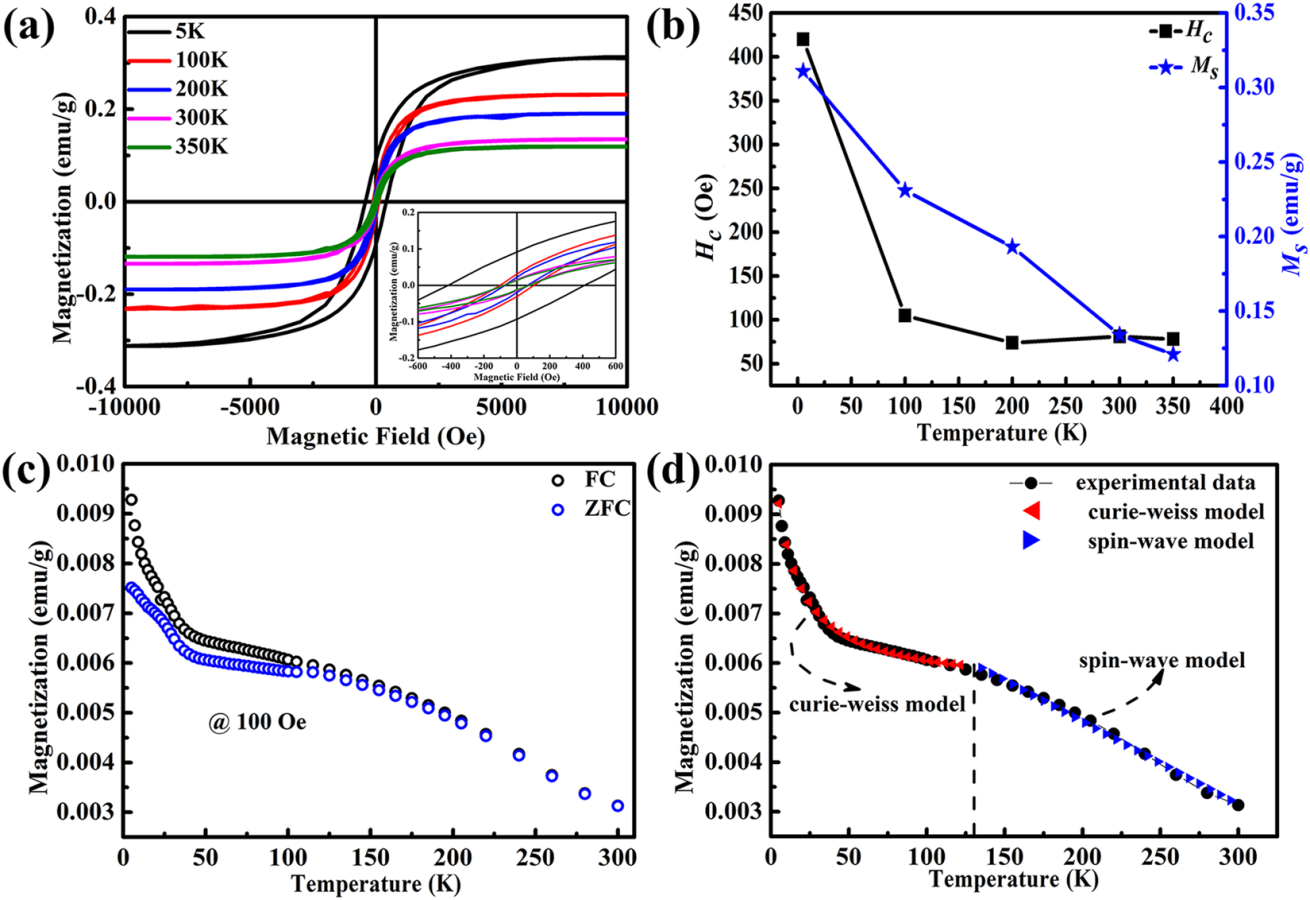
Magnetic properties of the as-prepared BCN nanosheets. (**a**) *M-H* curves measured at 5 K to 350 K; the inset shows the enlargement of the hysteresis loops in the range of −500 Oe < *H* < 500 Oe. (**b**) Relationship among *M*_*s*_, *H*_*c*_, and temperature. (**c**) Temperature dependen**c**e of field-cooled (FC, black open circles) and zero-field-cooled (ZFC, blue open circles) magnetization at 100 Oe for the as-prepared BCN nanosheets. (**d**) Theoretical fitting for the FC curve by Curie-Weiss model for the low temperature region and spin-wave theory model for the high temperature region.

The changes of magnetization with the temperature are studied by applying a magnetic field in the range of −10 k Oe < *H* < 10 k Oe at temperature ranging from 5 K to 350 K (BCN-3). Temperature dependent of magnetization is demonstrated clearly in Fig. 4a. For a more detailed and direct display, enlargement of the hysteresis loops of the BCN nanosheets are provided in the inset of Fig. 4a. The corresponding *H*_*C*_ and *M*_*s*_ show a downward trend with the increase in temperature (Fig. 4b). When the temperature is higher than 200 K, the variation in *H*_*C*_ becomes flat as the temperature increases. Zero-field-cooled (*ZFC*) and field-cooled (*FC*) magnetization measurements are conducted with a SQUID magnetometer at temperature ranging from 5 K to 300 K. As the increase of temperature, the *ZFC* and *FC* curves decrease continually. For the low temperature region, the curve follows a simple Curie-Weiss model^41^, *M* (*T*) = *M*_0_ + *C*/(*T* − *θ*), where *M*_0_ = 0.13 *μ*_*B*_ */*C atom, C = 2.03 K *μ*_*B*_/C atom, and *θ* = −25.97 K are the fitting parameters; for high temperature region, the curve can be well fitted by a standard spin-wave model which leads to *M* (*T*) = *M* (0)(1 − *AT* ^3/2^ *)*, where *M* (0) and *A* are the fitting parameters, where *M* (0) = 0.20 *μ*_*B*_/C atom and *A* = 1.01 *×* 10^−4^ K^−3/2^. The fit to a 3D spin-wave model suggests a clear ferromagnetic behavior over the temperature range. The *T*_*c*_ obtained from the spin-wave theory in the high temperature region, *M* (*T*) = *M* (0) (1 − *AT* ^3*/*2^), *M* (0*)* = 0.20 *μ*_*B*_/C atom and *A* = 1.01 *×* 10^−4^ K^−3/2^, is about 463 K.

The *ZFC* curve always lies below the *FC* curve in the low temperature region show the Pauli or Curie-like behavior which indicating that local spins exist. This tendency can be easily distinguish from conventional spin-glass system where the *FC* curve overlap together with the *ZFC* curve and *ZFC*/*FC* curves will quickly reduce to near zero when the temperature just above *T*_*f*_. The mutual suppression of exchange interaction between magnetic moments can resist the influence of an external magnetic field. So, if spin-glass system exists, it is easy to find in the *M-H* curve because the magnetic moment of spin-glass system cannot be uniform no matter what the magnetic field is. However, as shown in Figs 3c and 4a, when the magnetic field reaches 8000 Oe, magnetization basically saturates. Furthermore, the featureless *ZFC*/*FC* curves show that secondary or tiny magnetic phase is absent^42^, this is consistent with the inductively coupled plasma-atomic (ICP) results in the subsequent discussion. The purity of the starting materials and BCN samples were considered to investigate the origin of the ferromagnetic order of the BCN nanosheets. The ICP results (Table SI), show less than 10 ppm of the transition metal impurities are detected. The *M*_*s*_ caused by transition metal impurities can be calculated no more than 1.3 × 10^−6^ emu/g, which is insufficient to affect the ferromagnetic signal of the as-prepared BCN nanosheets^42^. The XPS results also show that no magnetic impurities were detected in the as-prepared BCN nanosheets, as shown in Fig. 2a and Supplementary Fig. 2a. These results are in agreement with ICP results. On the other hand, our results show that the *FM* is still robust against oxidation treatment (Supplementary Fig. 7). Therefore, we believe that the observed *FM* must be intrinsic.

## Discussion

**Figure 5:**
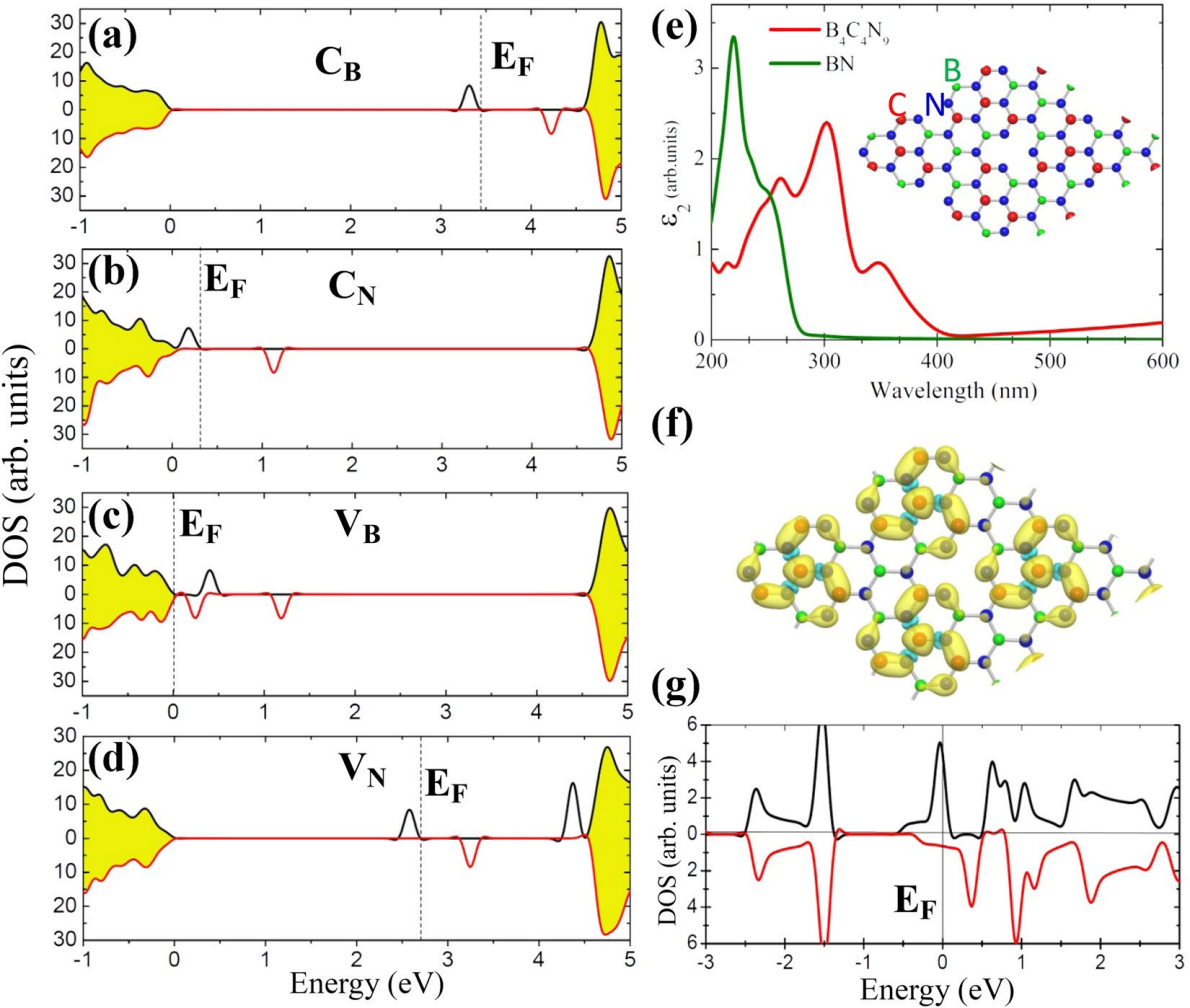
(**a**–**d**) Spin-resolved electron density of states (DOS) of BNNSs containing a C_B_, C_N_, V_B_, and V_N_ defect, respectively. The shadow area represents the states of BNNSs. The dashed lines indicate the positions of Fermi level. (**e**) The imaginary part of dielectric function ε_2_ (ω) which represents the light adsorption property calculated for pristine BNNSs and the C-doped BNNSs (B_4_C_4_N_9_) shown in the inset of this figure. (**f**) The isosurfaces of the spin-polarized electron density ∆ρ calculated from the difference between the electron densities of two spin channels: ∆ρ = ρ↑ − ρ↓. (**g**) The spin-resolved DOS of the B_4_C_4_N_9_ nanosheet. The energy at the Fermi level was set to zero.

To reveal the origins of band gap reduction and stable ferromagnetism in the BCN nanosheets, we performed first-principles calculations within density-functional theory (DFT) on the C-doped BNNSs. We employed 6 × 6 supercells (72 atoms) of BN doped with C atoms at different sites (referred to as C_B_ and C_N_). Two typical vacancy defects (V_B_ and V_N_) were also taken into account, since they are inevitable during the reactions. These four types of defects can generate local electronic states within the band gap of BNNSs at different energy regions, as shown in Fig. 5a–d. For the C_B_ and V_N_ defect, the local states are close to the conduction bands, whereas for the C_N_ and V_B_, they are close to the valence bands. With the increase of C concentration, the coupling between these local electronic states leads to dispersive electronic states which broaden the light adsorption region and reduce the optical band gap of the systems. To verify this mechanism, we built a BCN nanosheet containing four C_N_ and one V_B_ defects in a 3 × 3 BN supercell with a stoicheiometry of B_4_C_4_N_9_, corresponding to a C concentration of 23.5%, as shown in the inset of Fig. 5e. It is found that the light adsorption in the region of 300–400 nm is greatly improved compared with undoped BNNSs. The band gap narrowing is quite obvious in the electron density of states shown in Fig. 5g.

It is noteworthy that the electronic states induced by these four types of defects are spin-polarized. The spin-polarized states are energetically more preferable than the spin-unpolarized states by about 0.19 eV (for C_B_) and 0.21 eV (for C_N_), respectively. Each carbon dopant induces 1.0 *μ*_B_ local moments. However, the coexistence of these defects (e.g., C_B_/C_N_, C_B_/V_B_, C_N_/V_N_, and V_B_/V_N_) may quench the spin-polarization due the charge transfer between these local states. When a C_B_ and a C_N_ coexist in the 6 × 6 supercell, the electron spin-polarization is suppressed completely. We calculated the formation energies (*E*_*Form*_) of C_B_ and C_N_ using the formula: *E*_*Form*_ = *E*_*C-BN*_ + *μ*_*B/N*_ − *E*_*BN*_ − *μ*_*C*_ where *E*_*C-BN*_ and *E*_*BN*_ represent the total energies of BN monolayer with and without C_N_ (or C_B_), *μ*_*B*_, *μ*_*N*_ and *μ*_*C*_ are the chemical potentials calculated from bulk B crystal, N_2_ molecule and graphene, respectively. Unlike the cases of V_B_ and V_N_ in BN monolayer^43^, the both C_B_ and C_N_ are set to neutral charge states. It is found that the energy required to generate a C_B_ is lower than that requited to generate a C_N_ by about 1.66 eV per C dopant. This implies that the C dopants prefer to replace B atoms rather than N atoms in the BCN nanosheets, in good consistence with the XPS and XANES results where C−B bonds were not detected. The asymmetric C substitution is quite crucial for the ferromagnetism in the BCN nanosheets.

Taking the B_4_C_4_N_9_ nanosheet as an example, the ground state is spin-polarized with spin density residing in the region near the C dopants, as shown in Fig. 5f. The spin-polarized nature is more obvious in the DOS shown in Fig. 5g. Although there are four C_B_ defects in one unit cell, the total magnetic moments are only about 1.0 *μ*_B_, due to the quenching effect of V_B_ defect. This is consistent with the experimental result that saturation magnetic moments of the samples are about 0.12 *μ*_B_ per carbon dopant. Since the C−B bond was not detected from XPS and XANES, the spin quenching effect arises mainly from the V_B_ defects. We also checked the stability of the ferromagnetism from first-principles. Starting from different initial spin alignments, self-consistent calculations gave two magnetic ordering. One is ferromagnetic shown in Fig. 5f. Another is a nonmagnetic state with a zero net magnetic moment. The ferromagnetic ordering is energetically more favorable than the nonmagnetic one, convincing the stability of the ferromagnetism in the BCN nanosheets.

In summary, by means of a multi-step strategy, we have prepared metal-free 2D ternary BCN nanosheets. Those metal-free BCN nanosheets exhibit tunable band gap (5.5 eV to 2.6 eV) and intrinsic ferromagnetism (*T*_*c*_ ≈ 463 K) simultaneously by varying substitutional occupation of C concentration for the first time. C *K*-edge XANES spectra unambiguously validate the substitution of C for B in the as-prepared BCN nanosheets. Although the electron spin-polarization is suppressed completely when a C_B_ and a C_N_ coexist in the 6 × 6 supercell, first-principle calculations also demonstrate the synergetic effect between band gap engineering and intrinsic ferromagnetism. This straightforward strategy for controllable preparation of band gap engineering metal-free 2D ternary BCN nanosheets with magnetic will not only inspire extensive interests about manipulating ferromagnetism in metal-free system via 2D strategy but also provide a new candidate for the next-generation miniature spintronic devices and optical fields applications.

## Methods

### Materials

Hexagonal boron nitride (*h*-BN) was purchased from Alfa Aesar. Flake graphite and ethanol were of analytical reagent grade and purchased from Sinopharm Chemical Reagent Co. Ltd. (Shanghai). All reagents were used as received.

### Sample preparation

BNNSs and graphite were exfoliated by metallic sodium (0.5 g). The BCN nanosheets are synthesized by embedding C atoms into the lattice of BNNSs. A controlled heating rate of 5 °C/min was used and the quartz boat held at 850 °C for 1 h. Then they were cooled down naturally. The whole procedure was conducted under constant nitrogen flow. After cooling to room temperature, the quartz boat was putted in a muffle furnace. The quartz boat was heated to 600 °C with a controlled heating rate of 10 °C/min and held at this temperature for 4 h. A more detailed description of the experimental procedures is provided in the supporting information (S2).

### Characterization

The morphologies of the samples were investigated by SEM (S4800, Japan), TEM (JEM-2100F, Japan), AFM (Veeco dimension V, USA), XRD (D8 Advance, Germany), UV-vis diffuse reflectance spectra (Shimadzu UV2550, Japan), XPS (Thermo ESCALAB 250, USA), inductively coupled plasma-atomic (ICP) mass spectrometry (Atomscan Advantage, Germany), superconducting quantum interference device (Quantum Design MPMS–XL, USA), X-ray absorption near-edge structure (Beamline of BL12B of Nation al Syncrotron Radiation Laboratory, China), Theoretical calculations (National Super Computing Centre in Jinan, China).

## Electronic supplementary material

Supplementary Information
