# Supplementary material for: Metal-free Ternary BCN Nanosheets with Synergetic Effect of Band Gap Engineering and Magnetic Properties
Source: Sci Rep. 2017 Jul 26;7:6617. doi: 10.1038/s41598-017-07143-6 (PMC5529436; doi:10.1038/s41598-017-07143-6)
Supplement: Supplementary file 1 — Supplementary Information [file 41598_2017_7143_MOESM1_ESM.pdf]

Metal-free Ternary BCN Nanosheets with Synergetic Effect of Band Gap Engineering and Magnetic Properties

Changlong Sun[1,+], Fukun Ma[1,+], Liang Cai[2,+], Aizhu Wang[3], Yongzhong Wu[1], Mingwen Zhao[3,*], Wensheng Yan[2,*], Xiaopeng Hao[1,*]

[1]State Key Lab of Crystal Materials, Shandong University, 250100, Jinan, Shandong, P. R. China.

[2]National Synchrotron Radiation Laboratory, University of Science and Technology of China, 230029, Hefei, Anhui, P. R. China.

[3]Department of Physics, Shandong University, 250100, Jinan, Shandong, P. R. China.

Correspondence and requests for materials should be addressed to M. W. Z. (email: zmw@sdu.edu.cn); W. S. Y. (email: ywsh2000@ustc.edu.cn); X. P. H. (email: xphao@sdu.edu.cn).

[+]These authors contributed equally to this work.

Contents

S1 Structure and property characterization

S2 Experimental section

S3 Photographs of BNNSs and exfoliated graphite ethanol solution with the irradiation of a laser beam

S4 X-ray photoelectron spectroscopy (XPS) of BNNSs, BCN nanosheets

S5 X-ray powder diffraction (XRD) patterns of the BCN nanosheets

S6 Comparison of the Fourier Transform Infrared Spectroscopy (FTIR) of the as-prepared BCN nanosheets and BNNSs

S7 Optical images of BNNSs, exfoliated graphite and BCN nanosheets

S8 Absorption and magnetic properties of BNNSs, exfoliated graphite and BCN nanosheets

S9 Concentrations of transition metals Fe, Co, Ni and Mn in the pristine material and as-prepared BCN

S10 Room temperature *M-H* curves of BCN nanosheets before and after oxidation treatment.

S1 Structure and property characterization

The field emission scanning electron microscopy images were taken on a Hitachi S-4800 scanning electron microscope (SEM). Energy-dispersive spectroscopy (EDS) elemental scans were performed on the same instrument equip with detector (7593-H, Horiba). The transmission electron microscopy (TEM) was carried out on a JEM-2100F field emission electron microscope at an acceleration voltage of 200 kV. The high-resolution TEM (HRTEM), and corresponding energy-dispersive spectroscopy (EDS) mapping analyses were performed on a JEOL JEM-ARF200F TEM/STEM. X-ray diffraction (XRD) spectra were collected by a Bruker diffractometer (D8 Advance) with Cu Kα radiation (λ = 1.5418 Å). UV-vis diffuse reflectance spectra (DRS) results were obtained with a Shimadzu UV2550 recording spectrophotometer equipped with an integrating sphere from 200 to 800 nm ($BaSO_4$ was used as a reference). Atomic force microscopy (AFM) study in the present work was performed by means of Veeco DI Nanoscope MultiMode V system. X-ray photoelectron spectra (XPS) were acquired on the Thermo ESCALAB 250 with Al Kα radiation (1486.8 eV) as the excitation source. The inductively coupled plasma-atomic (ICP) mass spectrometry was carried out at plasma atomic emission spectrometry (Atomscan Advantage). The magnetization was characterized by a superconducting quantum interference device (SQUID, quantum design MPMS XL) magnetometer with a temperature range of 5 - 350K and applied field range of −10k to 10k Oe. The X-ray absorption near-edge structure data were collected at beamline BL12B of Nation al Syncrotron Radiation Laboratory (NSRL, China). Theoretical calculations are performed in the National Super Computing Centre in Jinan.

S2 Experimental section

Materials

Hexagonal boron nitride (*h*-BN) was purchased from Alfa Aesar. Flake graphite and ethanol were of analytical reagent grade and purchased from Sinopharm Chemical Reagent Co. Ltd. (Shanghai). All reagents were used as received.

Preparation of the overlapped mixture of BNNSs and exfoliated graphite

Under the protection of nitrogen, 0.5 g metallic sodium was added to a clean conical flask with a mixture of 0.1 g *h*-BN powder and 0.1 g graphite powder. The conical flask was sealed and then maintained at 120 ℃ in a stirred condition for 24 h. After cooling to room temperature, 20 mL n-hexane was added to the conical flask. Then, 100 mL ethanol was cautiously added, dropwise, to the conical flask. (Because of the reaction of metallic sodium and ethanol is tempestuous, the instillment reagent order's priority cannot be reversed absolutely). The conical flask was sonicated for 10 min. The product was collected and washed with ethanol and deionised water repeatedly until the pH of the filtrate was close to neutral. Then the product was placed in a 500 mL beaker filled with ethanol. After the beaker was shaken several times, the dispersion was allowed to settle for 2 weeks to deposit the undispersed particles. The supernatant was then collected by filtration, then ~ 85 mg of overlapping mixture of BNNSs and exfoliated graphite was obtained for further use. The initial addition of graphite powder can be changed for requirements.

Synthesis of BCN nanosheets

As-prepared overlapping mixture powder was putted into a quartz boat. The quartz boat was put into a quartz tube with diameter of 80 mm. After that, the tube was purged under nitrogen for 30 min to get an inert atmosphere. Then the quartz boat was heated in a tube furnace under nitrogen atmosphere. A controlled heating rate of 5 ℃/min was used and the quartz boat held at 850 ℃ for 1 h. Then they were cooled down naturally. The whole procedure was conducted under constant nitrogen flow. After cooling to room temperature, the quartz boat was putted

in a muffle furnace. The quartz boat was heated to 600 ℃ with a controlled heating rate of 10 ℃/min and held at this temperature for 4 h.

Details of XANES Calculations

To achieve convergence of calculation, a cluster consisting of 178 atoms was used for model structure. The total scattering potentials including a fully relaxed core-hole were obtained iteratively, by successive calculations of the potential until self-consistency was reached. Based on this scattering potential, the final states of the excited photoelectron were then calculated. The Hedin-Lundqvist model of exchange potential with a 0.2 eV shift and additional broadening of 0.1 eV was used to give a closest match between the simulated and experimental spectra.

Details of first-principles calculations

The first-principles calculations reported in this work were performed in the framework of density-functional theory (DFT) using the Vienna *ab initio* simulation package known as VASP.[2-4] The electron-electron interactions were treated within a generalized gradient approximation (GGA) in the form of Perdew-Burke-Ernzerhof (PBE) for the exchange-correlation functional[4]. The electron wavefunctions were expanded by plane-waves with an energy cutoff of 600 eV. The electron-ion interaction was described by projector-augmented wave (PAW) potentials.[6, 7] The supercells are repeated periodically on the *x*-*y* plane while a vacuum region of about 15 Å. The Brillouin zone (BZ) integration was sampled on a grid of 5 × 5 × 1 k-points for the large supercell (6 × 6), while for small-size suprecell (3 × 3), the k-points mesh is 11 × 11 × 1. Structural optimizations were carried out using a conjugate gradient (CG) method until the remaining force on each atom is less than 0.01 eVÅ^{-1}.

S3 Photographs of BNNSs and exfoliated graphite ethanol solution with the irradiation of a laser beam

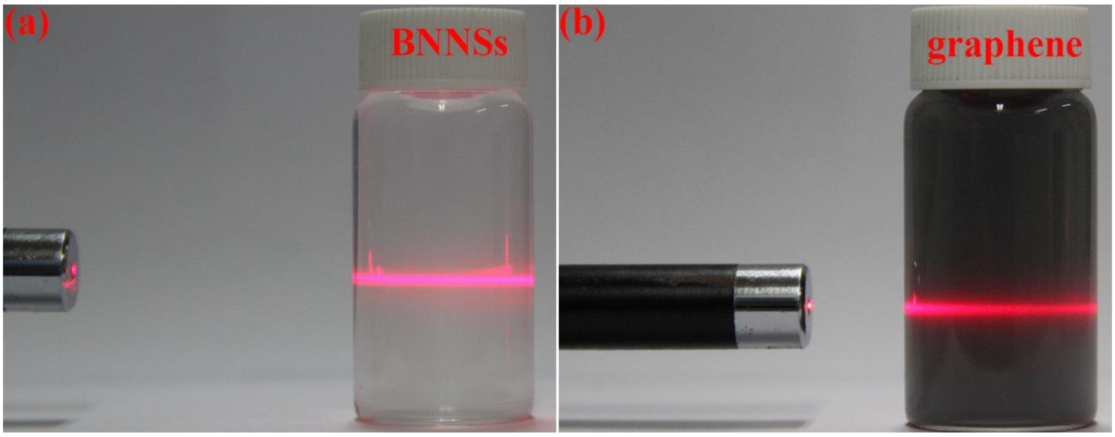

Fig. S1. Photographs of (a) BNNSs ethanol solution (left) and (b) exfoliated graphite ethanol solution (right) with the irradiation of a laser beam from the left.

The dispersibility of the as-prepared BNNSs and exfoliated graphite are studied by dispersing in ethanol solution. As shown in Fig. S1, BNNSs and exfoliated graphite can be exfoliated well by liquid phase exfoliation and preserved for more than three months, respectively. When the as-prepared BNNSs and exfoliated graphite dispersions are illuminated using a red laser, the Tyndall effect, a signature of colloidal dispersions, is observed.

S4 X-ray photoelectron spectroscopy (XPS) of BNNSs, as-prepared BCN nanosheets and the change of high-resolution XPS spectrum of N1s recorded from BCN nanosheets and BNNSs

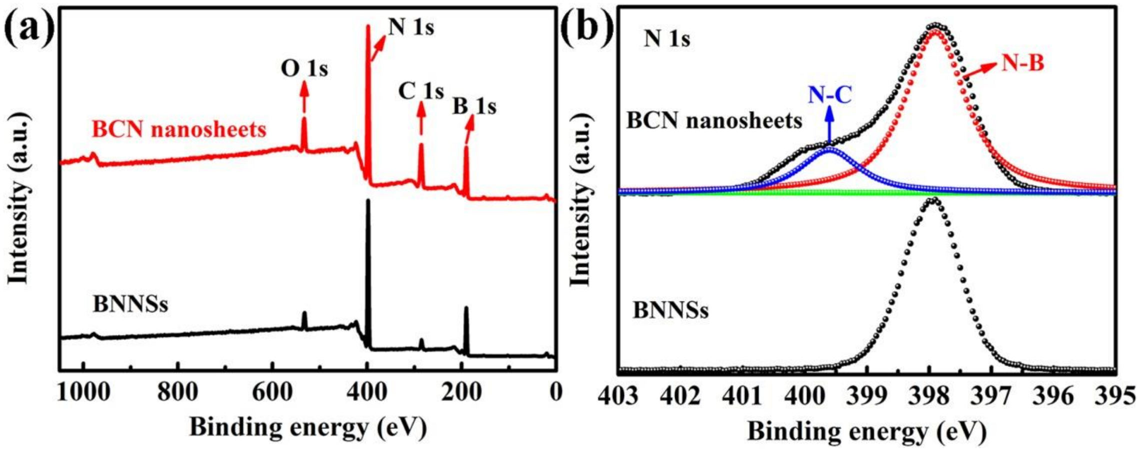

Fig. S2. (a) XPS spectra showing the binding energies of O, C, N, and B of as-prepared BCN nanosheets and BNNSs (b) the change of high-resolution XPS spectrum of N1s recorded from BCN nanosheets and BNNSs

As shown in Fig. S2a, the XPS spectra present the detailed differences of the as-prepared BNNSs and BCN nanosheets. There is a distinct difference in XPS spectra between BNNSs and BCN nanosheets. Compared with the carbon in BCN nanosheets, the binding energy of C in BNNSs is small and it was mainly caused by contamination. At the same time, high-resolution XPS spectrum of N1s recorded from BCN nanosheets and BNNSs are shown in Fig. S2b, the different composition and construction between the as-prepared BNNSs and BCN nanosheets can be clearly demonstrated.

S5 X-ray powder diffraction (XRD) patterns of the as-prepared BCN nanosheets

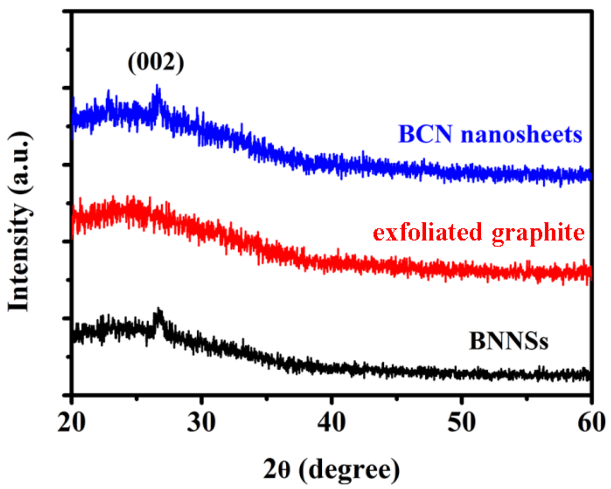

Fig. S3. X-ray diffraction patterns of the as-prepared BCN nanosheets, exfoliated graphite and BNNSs.

The XRD patterns of the exfoliated graphite and BNNSs are also presented in Fig. S3. The weak (002) peak of the BCN nanosheets suggests the exposure of the (001) facets of the BN matrix, consistent with the HRTEM results. The puny and broaden peaks indicated the ultrathin nature of the as-prepared BNNSs, exfoliated graphite, and BCN nanosheets. The similar puny and broaden XRD peaks have also been observed with samples prepared using the same spin-coating method.[8]

S6 Comparison of the Fourier Transform Infrared Spectroscopy (FTIR) of the as-prepared BCN nanosheets and BNNSs

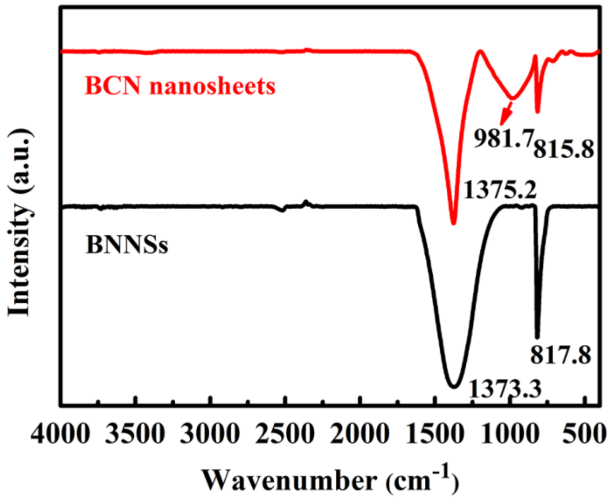

Fig. S4. FTIR spectra of the as-prepared BCN nanosheets and BNNSs.

Fig. S4 shows the FTIR spectra of the BNNSs and the as-prepared BCN nanosheets. Two typical sharp absorption peaks of the BNNSs were observed at 1373.3 and 817.8 cm^{-1} (caused by the in-plane B-N stretching vibration and the B-N-B out-of-plane bending vibration respectively). There are additional peak located at 981.7 cm^{-1} in the structure of the as-prepared BCN nanosheets, which suggest the C-N bonds in the structure.[9]

S7 Optical images of BNNSs, exfoliated graphite and as-prepared BCN nanosheets

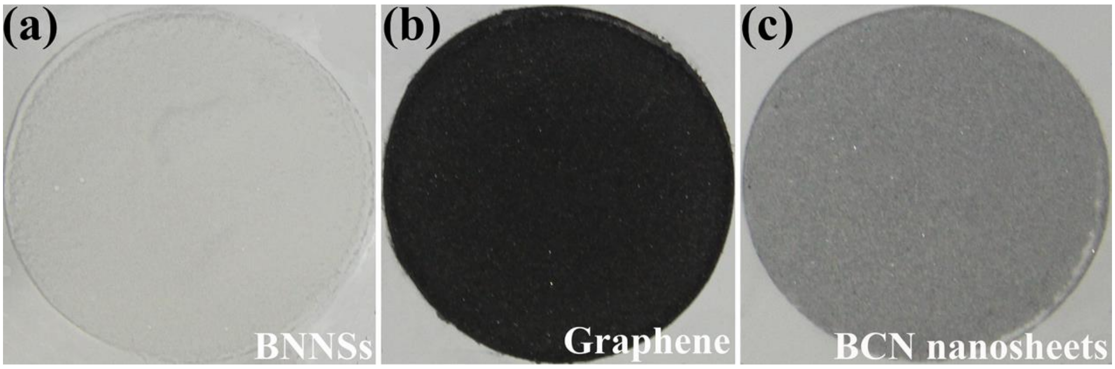

Fig. S5. Optical images of BNNSs, exfoliated graphite and as-prepared BCN nanosheets

Fig. S5 shows optical images of the as-prepared BNNSs, exfoliated graphite and BCN nanosheets, respectively. The color of the as-prepared BCN nanosheets is different from that of raw materials.

S8 Absorption and magnetic properties of BNNSs, exfoliated graphite and BCN nanosheets

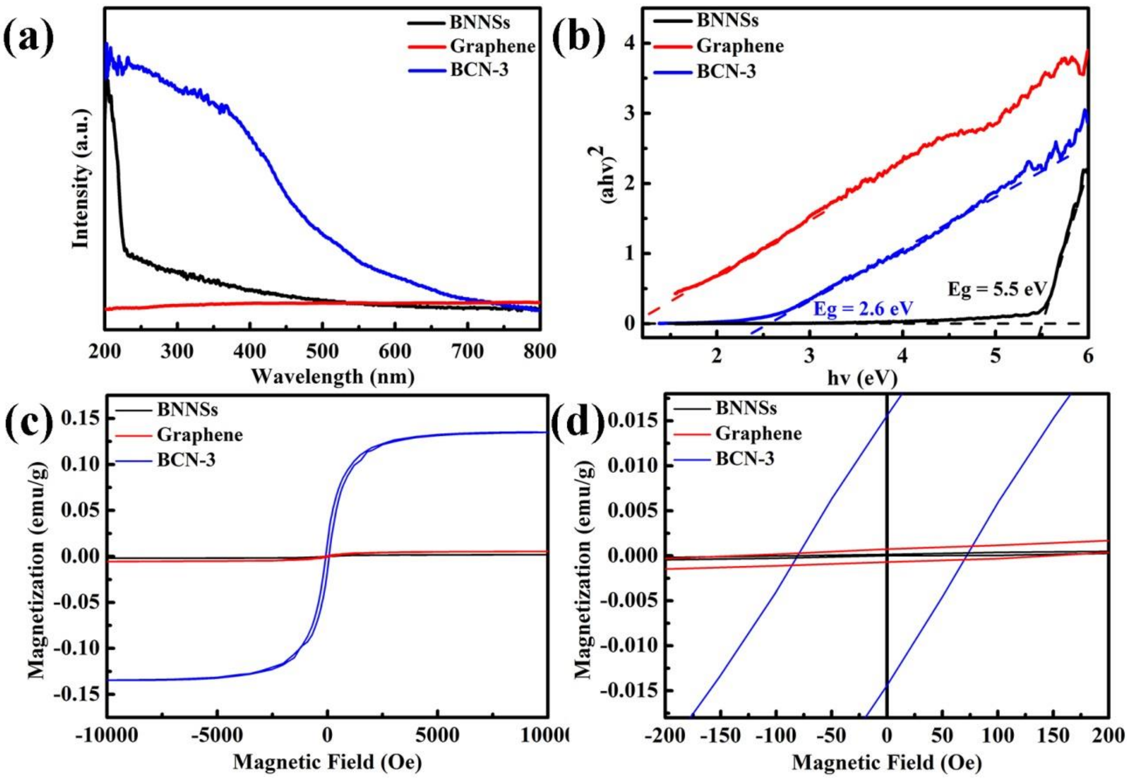

Fig. S6. Absorption and magnetic properties of the as-prepared BNNSs, exfoliated graphite and BCN nanosheets. (a) UV−vis DRS results of BNNSs, exfoliated graphite and BCN nanosheets. (b) Tauc plot of BNNSs, exfoliated graphite and BCN nanosheets. (c) Magnetization *vs*. magnetic field (*M*-*H*) of BCN nanosheets measured at room temperature (300K) relative to the exfoliated graphite and BNNSs. (d) Zoom-in plots showing the remanence magnetizations.

As shown in Fig. S6a, the absorption property of the as-prepared BCN nanosheets is different from exfoliated BNNSs and exfoliated graphite. The absorption spectrum of the BNNSs sample shows the absorption edge at 225 nm, which corresponds to an optical band gap of 5.5 eV. The absorption spectrum of the BCN nanosheets sample shows the absorption edge at 480 nm, which corresponds to an optical band gap of 2.6 eV. This indicated the doping C concentration has effect on the optical band gap of the as-prepared BCN nanosheets as shown in Fig. S6b. A similar phenomenon has occurred in the BCN prepared by pyrolysis

method.[8] We checked room-temperature magnetic property of the precursors. As shown in Fig. S6c, the *M−H* curve of the pristine exfoliated BNNSs and exfoliated graphite indicate the weak room-temperature *FM* behavior, close to the previous reports, different from the nonmagnetic property of bulk BN and graphite.[10, 11]

S9 Concentrations of transition metals Fe, Co, Ni and Mn in the pristine material and as-prepared BCN

Table S1. Concentrations of transition metals in the pristine material and as-prepared BCN

	Fe [ppm]	Co [ppm]	Ni [ppm]	Mn [ppm]
BN	1.7864	Not detected	0.1192	0.0080
graphite	3.5304	Not detected	0.084	1.0496
BCN-1	1.4928	Not detected	0.1048	Not detected
BCN-2	1.6527	Not detected	0.0431	0.0132
BCN-3	1.8751	Not detected	Not detected	Not detected

Magnetic property can be greatly influenced by the transition metal impurities, so it is necessary to detect the impurities of the starting materials and as-prepared samples by inductively coupled plasma-atomic (ICP) before measuring the magnetic of the as-prepared BCN nanosheets. From the data listed in the table I, it can be seen that the total amount of possible magnetic impurities of Fe, Co, Ni and Mn are estimated at less than 10 ppm.

S10 Room temperature *M-H* curves of BCN nanosheets before and after oxidation treatment.

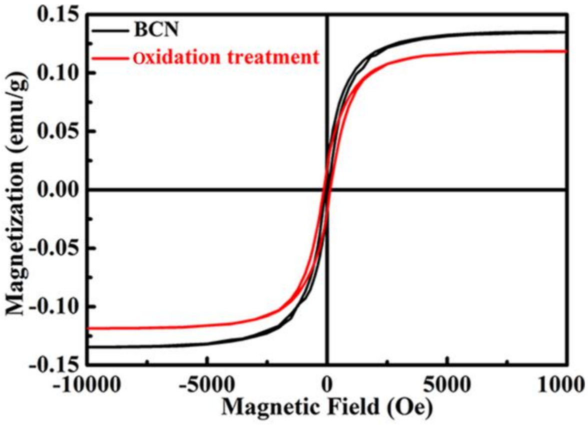

Fig. S7. Room temperature *M-H* curves of BCN nanosheets before and after oxidation treatment.

On the other hand, stable of *FM* is another important thing to consider. The as-prepared BCN nanosheets were burned for 2 h in O_2 atmosphere at 923 K in a muffle furnace. Our results show that the *FM* is still robust against oxidation treatment. In contrast to the case reported in a previous report that the *FM* was removed completely from C-doped BNNSs by oxidation,[12] the M_s of the as-prepared BCN nanosheets only decrease slightly after the oxidation treatment, Fig. S7. This confirms that the C dopants were really incorporated into the BN lattice instead of chemically adsorbed on the BNNSs.

References

1. Perdew, J. P. and A. Zunger, Self-interaction correction to density-functional approximations for many-electron systems. *Phys. Rev. B* **23**, 5048-5079 (1981).
2. Coleman, J. N. *et al.* Two-Dimensional Nanosheets Produced by Liquid Exfoliation of Layered Materials. *Science* **331**, 568-571 (2011).
3. Kresse, G. and Furthmüller, J. Efficiency of ab-initio total energy calculations for metals and semiconductors using a plane-wave basis set. *Comp. Mater. Sci.* **6**, 15-50 (1996).
4. Kresse, G. and Furthmüller, J. Efficient iterative schemes for ab initio total-energy calculations using a plane-wave basis set. *Phys. Rev. B* **54**, 11169-11186 (1996).
5. Perdew, J. P., Burke, K., and Ernzerhof, M. Generalized Gradient Approximation Made Simple. *Phys. Rev. Lett.* **77**, 3865-3868 (1996).
6. Blöchl, P. E. Projector augmented-wave method. *Phys. Rev. B* **50**, 17953-17979 (1994).
7. Kresse, G. and Joubert, D. From ultrasoft pseudopotentials to the projector augmented-wave method. *Phys. Rev. B* **59**, 1758-1775 (1999).
8. Huang, C. *et al.* Carbon-doped BN nanosheets for metal-free photoredox catalysis. *Nat. Commun.* **6**, 7698 (2015).
9. Lotsch, B. V. *et al.* Unmasking Melon by a Complementary Approach Employing Electron Diffraction, Solid-State NMR Spectroscopy, and Theoretical Calculations-Structural Characterization of a Carbon Nitride Polymer. *Chem. Eur. J.* **13**, 4969–4980 (2007).
10. Wang, Y. *et al.* Room-Temperature Ferromagnetism of Graphene. *Nano lett.* **9**, 220-224 (2009).
11. Si, M. S. *et al.* Intrinsic ferromagnetism in hexagonal boron nitride nanosheets. *J. Chem.Phys.* **140**, 204701 (2014).

12. Zhao, C. *et al.* Carbon-Doped Boron Nitride Nanosheets with Ferromagnetism above Room Temperature. *Adv. Funct. Mater.* **24**, 5985-5992 (2014).
